# SGRTmreg: A Learning-Based Optimization Framework for Multiple Pairwise Registrations

**DOI:** 10.3390/s24134144

**Published:** 2024-06-26

**Authors:** Yan Zhao, Jiahui Deng, Qinghong Gao, Xiao Zhang

**Affiliations:** 1 School of Information Science and Technology, Northwest University, Xi’an 710127, China; yanzhaonancy@gmail.com (Y.Z.); dengjiahui@stumail.nwu.edu.cn (J.D.); 2 Department of Creative Technology, Bournemouth University, Poole BH12 5BB, UK; s5222110@bournemouth.ac.uk

**Keywords:** mathematical optimization, point cloud registration, supervised learning, deep learning

## Abstract

Point cloud registration is a fundamental task in computer vision and graphics, which is widely used in 3D reconstruction, object tracking, and atlas reconstruction. Learning-based optimization and deep learning methods have been widely developed in pairwise registration due to their own distinctive advantages. Deep learning methods offer greater flexibility and enable registering unseen point clouds that are not trained. Learning-based optimization methods exhibit enhanced robustness and stability when handling registration under various perturbations, such as noise, outliers, and occlusions. To leverage the strengths of both approaches to achieve a less time-consuming, robust, and stable registration for multiple instances, we propose a novel computational framework called **SGRTmreg** for multiple pairwise registrations in this paper. The SGRTmreg framework utilizes three components—a **S**earching scheme, a learning-based optimization method called **G**raph-based **R**eweighted discriminative optimization (GRDO), and a **T**ransfer module to achieve **m**ulti-instance point cloud **reg**istration.Given a collection of instances to be matched, a template as a target point cloud, and an instance as a source point cloud, the searching scheme selects one point cloud from the collection that closely resembles the source. GRDO then learns a sequence of regressors by aligning the source to the target, while the transfer module stores and applies the learned regressors to align the selected point cloud to the target and estimate the transformation of the selected point cloud. In short, SGRTmreg harnesses a shared sequence of regressors to register multiple point clouds to a target point cloud. We conduct extensive registration experiments on various datasets to evaluate the proposed framework. The experimental results demonstrate that SGRTmreg achieves multiple pairwise registrations with higher accuracy, robustness, and stability than the state-of-the-art deep learning and traditional registration methods.

## 1. Introduction

Point cloud registration has been actively studied in computer vision and graphics [1,2,3,4,5,6], and most studies mainly focus on pairwise registration [7]. The primary objective of pairwise registration is to estimate the transformation parameters that align a source point cloud to a target point cloud. However, there is a multi-instance point cloud registration scenario, where multiple instances are aligned to a fixed template via multiple pairwise registrations. Multiple pairwise registrations make the existing registration methods more time-consuming, especially for the traditional methods with the estimation of the Hessian or inverse Hessian matrix, applying them to the registration of the point clouds obtained from LiDAR with variations in perturbations and point density, demanding high computational capacity and processing time [8].

Learning-based optimization methods [9,10,11] efficiently learn gradient directions without calculating Jacobian or Hessian matrices, which is relatively less time-consuming. Additionally, they adopt a model- or feature-driven approach to learn regressors from data to mimic gradients, resulting in heightened stability and robustness in the registration with various perturbations. However, the current approach restricts the learned regressors to training and testing an individual model, lacking flexibility and efficiency for multiple pairwise registrations.

Deep learning methods [12,13,14,15,16,17,18,19,20,21] significantly enhance point cloud registration by automatically extracting features and estimating transformations with learned regressors based on point correspondences. Their data-driven nature bestows them with flexibility, enabling the registration of unseen point clouds. However, this reliance on data can potentially impact registration performance, particularly when confronted with diverse perturbations such as noise, outliers, and occlusions.

To enhance the efficiency, stability, and robustness of multiple pairwise registrations, we introduce SGRTmreg, a new computational framework. Given a collection of point clouds, a source point cloud, and a target point cloud, the process for SGRTmreg to achieve registration unfolds in three steps: (1) Selecting a point cloud similar to the source from the collection based on graph structure, coordinates, node importance, and normal vectors via a searching scheme. (2) Learning regressors from the source using the Graph-based Reweighted Discriminative Optimization (GRDO) method by registering the source to the target. GRDO encodes features and learns regressors from key points in graph structures, reducing memory storage and computational costs. (3) Using the learned regressors to estimate the transformation from the selected point cloud to the target via a transfer module. Notably, the learned regressors possess the versatility to be employed in registering any other point clouds resembling the selected one.

We demonstrate the potential of SGRTmreg in multiple pairwise registrations on the ModelNet40 dataset and showcase the high performance of GRDO in registration under various perturbations on synthetic datasets, the WHU-TLS dataset [22] and the UWA dataset [23]. Our experimental results exhibit the accuracy and stability of SGRTmreg in multiple pairwise registrations, with GRDO surpassing advanced registration methods in robustness, accuracy, and stability. The contributions of this paper are the following:

- SGRTmreg achieves higher accuracy and robustness in the multiple pairwise registrations.
- GRDO outperforms advanced learning-based optimization methods in robustness, stability, and computational/storage efficiency.
- The proposed key points selection method retains detailed information compared to common downsampling approaches [24].

## 2. Related Work

### 2.1. Point Cloud Registration

Point cloud registration aligns two point clouds into a common coordinate system. The Iterative Closest Point (ICP) method [25] is widely used to find the optimal rigid transformation by iteratively minimizing the point cloud difference. Coherent Point Drift (CPD) [26] casts point cloud registration as the matching of Gaussian mixture models, which moves the Gaussian mixture model centroids coherently to preserve the topological structure of point clouds. Bayesian Coherent Point Drift (BCPD) [27] replaces the motion coherence theory in CPD with Bayesian inference. Both CPD and BCPD focus on point-to-point distance without considering local surface geometry. LSGCPD [28] incorporates varying levels of point-to-plane penalization alongside point-to-point penalization. TEASER++ [29] leverages estimation theory, geometry, graph theory, and optimization to register point clouds in the presence of large amounts of outlier correspondences. A scale-adaptive ICP method is introduced in [30] for aligning objects differing by rigid transformations (translations, rotations) and uniform scaling. QGORE [31] employs “rotation correspondence” to establish a one-point RANSAC for lower bound estimation and proposes geometric consistency voting for tight upper bound seeking, which is the first quadratic–time guaranteed outlier removal method for point cloud registration. These traditional methods approach point cloud registration as an optimization problem involving designing objective functions and function solutions. Objective functions are typically tailored to address the registration under specific perturbations, such as noises, outliers, and occlusions. Gradient-based methods are widely employed as function solvers, which often require approximations of the Hessian or inverse Hessian matrices, making it challenging to solve objective functions with a large number of parameters or high storage requirements.

To avoid calculating gradients, learning-based optimization methods utilize supervised sequential update methods to learn regressors emulating gradient directions. Ref. [30] uses regressors to update shape parameters based on image features. The Discriminative Optimization method (DO) [10] adopts the least-squares method to learn regressors mapped to the features of point clouds to estimate transformation parameters. The Reweighted Discriminative Optimization method (RDO) [11] designs an asymmetrical parameter treatment scheme to learn regressors. While learning-based optimization methods demonstrate robustness and stability in handling registrations with various perturbations, they are unable to register multiple point cloud pairs using the learned regressors from individual point clouds.

The success of deep learning techniques in image processing has been extended to point cloud registration. PointnetLK [16] utilizes the Lucas–Kanade algorithm [32] to estimate transformation on a global feature space. DCP [17] replaces the Lucas–Kanade algorithm with differentiable singular value decomposition. RPMNet [20] inputs point clouds and normals to extract features and then estimate point correspondences. RGM [21] transforms point clouds into graphs and calculates correspondences via a graph feature extractor. FMR [18] estimates transformation by minimizing a feature-metric projection error without seeking correspondences. DeepGMR [19] formulates registration as KL-divergence minimization between mixtures of Gaussians. SACF-Net [14] incorporates a novel feature interaction mechanism to enhance pointwise matching by leveraging both low-level geometric and high-level context-aware information. GeoTransformer [33] encodes pair-wise distances and triplet-wise angles to learn geometric features for registration, which ensures invariance to rigid transformations and enhances robustness in low-overlap scenarios. PAnet [34] proposes a point-attention-based multi-scale feature fusion network for partially overlapping point cloud registration. RoReg [35] utilizes oriented descriptors and estimated local rotations throughout the registration pipeline. It introduces a novel oriented descriptor, RoReg-Desc, which is employed for estimating the local rotations. GMCNet [36] employs a novel transformation-robust point transformer module to adaptively aggregate local features with respect to the structural relations, taking advantage of both handcrafted rotation-invariant features and noise-resilient spatial coordinates to estimate correspondences for full-range partial-to-partial point cloud registration. RIGA [37] develops descriptors with rotation-invariant and globally-aware methods to extract robust correspondences for registration. PointTr [38] employs a learnable geometric position update module and a deeper cross-attention module to automatically learn and capture the geometric structure and features among partial point clouds. The limitations of these methods are twofold: (1) performance drops significantly when applied to unseen point clouds with structural differences from the training data; (2) vulnerability to perturbations due to high data reliance. Nevertheless, deep learning methods provide greater flexibility, enabling training on large amounts of data and testing with any relevant data, a limitation of learning-based optimization methods.

In summary, learned-based optimization methods offer advantages over traditional registration methods by learning regressors directly from data without the need for designing objective functions or calculating gradient matrices. They also exhibit greater robustness compared to deep learning methods and are less dependent on data size. However, they may lack the flexibility of deep learning methods, as they solely rely on learned regressors for registering an individual point cloud pair. Given this, we develop a framework named SGRTmreg for multiple pairwise registrations, utilizing the core insight of learning-based optimization methods—supervised sequential update methods.

### 2.2. Supervised Sequential Update Methods

Learning-based optimization methods use supervised sequential update methods to learn regressors that mimic gradient directions, avoiding explicit gradient calculations. This is completed by learning a sequence of regressors that maps a feature vector to an update vector that points to the desired parameters. Here, we provide a brief review of supervised sequential update methods.

Dollár et al. [39] propose a cascaded pose regression to compute 2D object poses in images. Cao et al. [40] develop an explicit shape regression method for face alignment by learning a vectorial regression function. Tuzel et al. [41] present a learning-based tracking method combined with object detection, where a linear regression function represents the descent direction. Xiong et al. [9] learn a sequence of regressors to update shape parameters based on image features per iteration. Most supervised sequential update methods focus on image-based tracking and pose estimation. Vongkulbhisal et al. [10,42] propose DO as an extension of the supervised sequential update methods and apply DO in the 3D registration. Inspired by DO, Zhao et al. [11] introduce an asymmetrical parameter treatment scheme in the least squares method, and Deng et al. [43] develop a generative optimization method for non-rigid registration.

While these methods offer the advantage of not requiring gradient calculation, they suffer from a longer feature extraction time with increasing points, making the registration of dense point clouds infeasible. Additionally, they are commonly used for identical point cloud registration, wherein the test point cloud is generated by introducing a specific perturbation to a training point cloud, which is determined by the following updating criteria of regressors:

(1) $$x_{t+1}=x_{t}-D_{t+1}f\left(x_{t}\right).$$

Here, $f:R^{p}\to R^{f}$ is a function that encodes a feature of a point cloud, and $D_{t+1}\in R^{p\times f}$ is a regressor that regresses the feature $f\left(x_{t}\right)$ to an update vector. $x_{t+1}$ is the updating parameter vector for transformation estimation. The prerequisite for the learned regressors $D_{t+1}$ attained in the training stage being used to estimate the parameter vector $x_{t+1}$ of the test point cloud is that the features of training and test point clouds must be similar, or at the very least, possess the same dimensions. Accordingly, we devise a search scheme to select a point cloud similar to the target, ensuring the successful application of the learned regressor for the registration of the target model.

## 3. Methodology

In this section, we denote a collection of point clouds as P, a source point cloud as Q, and a target point cloud as M. SGRTmreg aims to utilize one sequence of regressors $D_{t+1}$ to register two point cloud pairs (〈Q,M〉 and 〈S,M〉), where S is the selected point cloud from P and is the most similar to Q. Note that if there is another point cloud $S^{\prime }$ similar to S, SGRTmreg can utilize $D_{t+1}$ to register $\langle S^{\prime },M\rangle$ as well.

**Figure 1:**
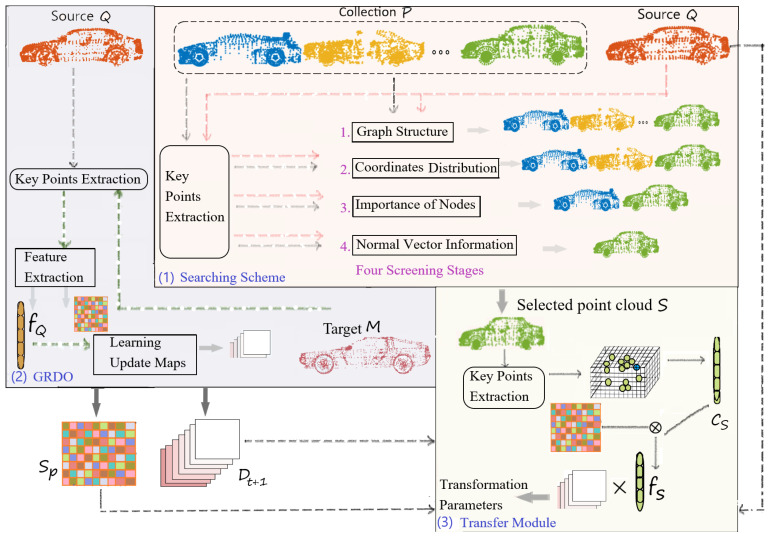
The framework of SGRTmreg includes three steps: (1) a searching scheme for selecting a similar point cloud S from a collection P for the source model Q by comparing the similarity of P and Q in four screen stages; (2) considering graph structure, coordinate distribution, and the importance of nodes and normal vector information, a learning-based optimization method called GRDO for learning a single sequence of regressors $D_{t+1}$ via the alignment of the source model Q and the target model M; (3) a transfer module for estimating the transformation between 〈S,M〉 by transferring the learned regressors $D_{t+1}$ to the selected model S. To reduce storage requirements and computation costs, each step works on the extracted key points.

The critical steps for SGRTmreg to achieve the registration of multiple point cloud pairs are: (1) Utilizing a searching scheme to select the point cloud S closely resembling the source point cloud Q from the collection P. (2) Learning the sequence of regressors $D_{t+1}$ by registering Q and M via the Graph-based Reweighted Discriminative Optimization (GRDO) method. (3) Applying $D_{t+1}$ in a transfer module to estimate the transformation parameters aligning S to M, as shown in Figure 1. Specifically, first, the searching scheme identifies the similar point cloud S by successively comparing the similarity of key points in the source point cloud Q with those in each point cloud in the collection P across four screening stages, considering graph structure, coordinate distribution, node importance, and normal vector information. Then, GRDO learns the sequence of regressors $D_{t+1}$ by aligning Q to M via the extracted feature $f_{Q}$ from the key points in Q. Last, the transfer module estimates the transformation from S to M by mapping the learned regressors $D_{t+1}$ to the feature $f_{S}$ of the key points in S.

### 3.1. Key Point Extraction

**Figure 2:**
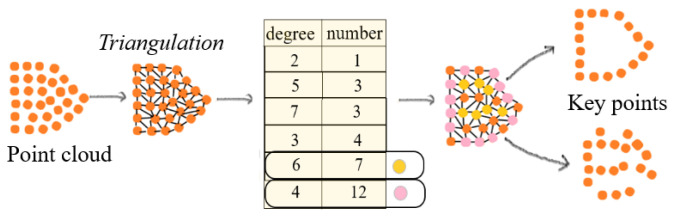
The process of key point extraction. The pink and orange are the extracted key points.

**Figure 3:**
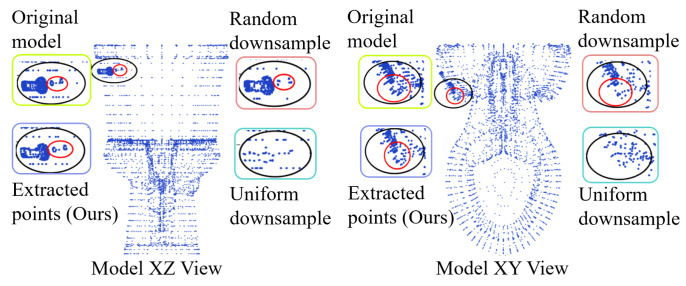
The comparison of our key point extraction approach with the random and uniform downsample methods. This figure shows the zoomed-in model from the XZ and XY perspectives. The dark circles show the difference. The red circles show the main detailed difference.

To reduce the storage requirement for designing features and learning regressors $D_{t+1}$ while cutting computational costs for GRDO, we design a key point extraction approach for downsampling point clouds. Figure 2 shows the process of key point extraction. Given a point cloud, Delaunay triangulation is applied to the top view (xy-view) of the point cloud to form a graph [44], where nodes represent vertices and edges represent connections between nodes. Then, the degrees of all nodes in the graph are counted. The degree of a node is the number of connections that it has to other nodes in the graph. Nodes with higher degrees have more connections, signifying their greater importance. Nodes connected by the non-shared edge between two triangles will be extracted as boundary points. The nodes whose degree has the most or the second most occurrence number and boundary points are selected as key points. Figure 3 shows that the proposed key point extraction approach reduces points while preserving detailed model information in contrast to the random and uniform downsample methods [24].

### 3.2. Searching Scheme

**Figure 4:**
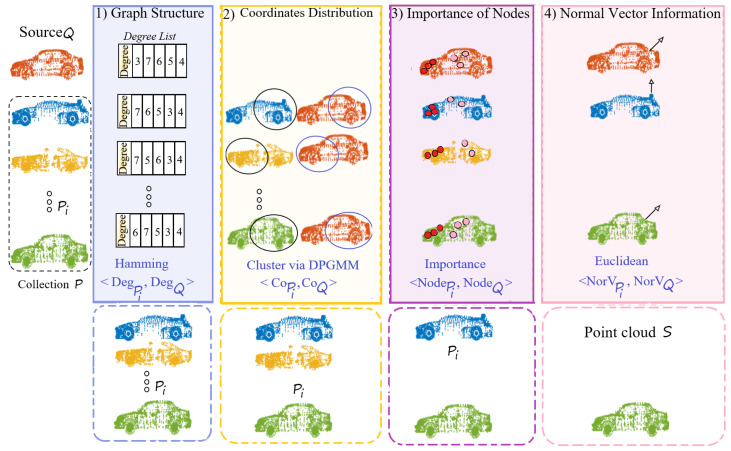
The structure of the searching scheme, including four screening stages, considering graph structure, coordinate distribution, the importance of nodes, and normal vector information. The dotted rectangle displays the candidate point clouds moving to the next screening stage. The points circled by blue and black are clustered in the same group. Red shows the nodes with the highest importance and pink with the lowest.

The searching scheme aims to identify the most similar point cloud S from the set P by comparing the similarity between each point cloud $P_{i}\in R^{N^{Pi}\times 3}$ and the target Q through four screening stages: (1) Measure the graph structure similarity between point cloud pairs $\langle P_{i},Q\rangle$ by employing the Hamming distance on their degree lists $\langle {\mathrm{Deg}}_{P_{i}},{\mathrm{Deg}}_{Q}\rangle$. (2) Measure the similarity of coordinate distribution $\langle {\mathrm{Co}}_{P_{i}},{\mathrm{Co}}_{Q}\rangle$ by clustering the mix of key points in $\langle P_{i},Q\rangle$ via the Dirichlet Process Gaussian Mixture Model (DPGMM) [45]. (3) Measure the similarity in the importance of graph nodes $\langle {\mathrm{Node}}_{P_{i}},{\mathrm{Node}}_{Q}\rangle$ using the Eigenvector centrality method [46]. (4) Measure the similarity in normal vectors $\langle {\mathrm{NorV}}_{P_{i}},{\mathrm{NorV}}_{Q}\rangle$ in Euclidean space. The point cloud $P_{i}$ passing these four screening stages will be chosen as the similar point cloud S, as shown in Figure 4.

#### 3.2.1. Similarity in Graph Structure

After converting a point cloud into a graph via the Delaunay triangulation in Section 3.1, the degree of nodes is initially used to sift through candidate point clouds. ${\mathrm{Deg}}_{P_{i}}\;=\;\left[de_{P_{i}}^{1},\ldots ,de_{P_{i}}^{j},\ldots \right]$ and ${\mathrm{Deg}}_{Q}\;=\;\left[de_{Q}^{1},\ldots ,de_{Q}^{j},\ldots \right]$ are the degree lists of $P_{i}$ and Q, respectively, where each element represents the degree of a node. We sort degrees based on their occurrences and ensure that the length of ${\mathrm{Deg}}_{P_{i}}$ matches that of ${\mathrm{Deg}}_{Q}$. If the length of ${\mathrm{Deg}}_{P_{i}}$ is larger, the degree with less occurrence will be removed. If it is shorter, ${\mathrm{Deg}}_{P_{i}}$ will be filled with 0.

(2) $$P_{De}^{i}=1-\frac{d_{H}\left({\mathrm{Deg}}_{P_{i}},{\mathrm{Deg}}_{Q}\right)}{L}.$$

where $d_{H}$ is the *Hamming* distance. The Hamming distance between ${\mathrm{Deg}}_{P_{i}}$ and ${\mathrm{Deg}}_{Q}$ is the count of differing elements at corresponding positions. *L* is the length of ${\mathrm{Deg}}_{Q}$. $P_{i}$ will enter the second stage as a candidate if the similarity $P_{De}^{i}$ is larger than β. $\beta \in \left(0.5,1\right)$ will always be set manually.

#### 3.2.2. Similarity in Coordinate Distribution

The coordinate distribution reflects the rough shape of a point cloud. The similarity in coordinate distributions $\langle {\mathrm{Co}}_{P_{i}},{\mathrm{Co}}_{Q}\rangle$ is measured by applying DPGMM to cluster the mixture of key points in $\langle P_{i},Q\rangle$. Suppose the mixture has been divided into *K* clusters $C_{k}=\left\{C_{P_{i}}^{k},C_{Q}^{k}\right\}$, $k\in \left\{1,\ldots ,K\right\}$. $C_{P_{i}}^{k}$ represents key points from $P_{i}$, and $C_{Q}^{k}$ represents key points from Q, both clustered in $C_{k}$, with dimensions $R^{N_{P_{i}}^{k}\times 3}$ and $R^{N_{Q}^{k}\times 3}$.

(3) $$R_{P_{i}}=\left[\frac{N_{P_{i}}^{1}}{N^{P_{i}}},\ldots ,\frac{N_{P_{i}}^{K}}{N^{P_{i}}}\right].$$

(4) $$R_{Q}=\left[\frac{N_{Q}^{1}}{N^{Q}},\ldots ,\frac{N_{Q}^{K}}{N^{Q}}\right].$$

The elements in $R_{P_{i}}$ and $R_{Q}$ depict the proportion of $C_{P_{i}}^{k}$ in ${\mathrm{Co}}_{P_{i}}$ and that of $C_{Q}^{k}$ in ${\mathrm{Co}}_{Q}$. $N^{P_{i}}$ and $N^{Q}$ are the number of key points in $P_{i}$ and Q, respectively.

(5) $$\tau_{P_{i}}=\sum_{k=1}^{K}\left(k\cdot \delta \left(\frac{N_{P_{i}}^{k}}{N^{P_{i}}}-\max\left(R_{P_{i}}\right)\right)\right).$$

(6) $$\tau_{Q}=\sum_{k=1}^{K}\left(k\cdot \delta \left(\frac{N_{Q}^{k}}{N^{Q}}-\max\left(R_{Q}\right)\right)\right).$$

where δ is the *Dirac delta* function [47]. Equations (5) and (6) illustrate that $C_{\tau_{P_{i}}}$ and $C_{\tau_{Q}}$ cluster most of the points in $P_{i}$ and Q. If $\tau_{P_{i}}=\tau_{Q}$, it implies that $P_{i}$ and Q have similar coordinate distributions (as shown in the cluster circled in Figure 4), and $P_{i}$ will be moved onto the next round. Please note that if ${\mathrm{Co}}_{Q}$ is equally divided, $P_{i}$ will also enter the next round as a candidate.

#### 3.2.3. Similarity in the Importance of Nodes

After sifting out point clouds with shapes similar to source Q, the similarity in internal structure is considered for further screening. The internal structure is revealed through node importance, quantified using the eigenvector centrality method [46]. The eigenvector centrality method evaluates the importance of a node based on how important the nodes in contact with it are: the higher the latter is, the higher the former becomes. Assuming the key points in source Q have been converted to the graph $G_{Q}$ with an adjacency matrix A, the absolute value of its principal eigenvector serves as the score for all nodes, revealing the eigenvector centrality of the graph $G_{Q}$ [46]. The eigenvector centrality of $P_{i}$ can be attained in the same way. If the average score of all nodes in $P_{i}$ is closest to that of Q, $P_{i}$ becomes a candidate for the next screening stage. To prevent eliminating the most similar point cloud during this screening, we relax the number of candidates entering the next stage to $\beta^{\prime }$.

#### 3.2.4. Similarity in Normal Vectors

The similarity in normal vectors is the final criterion for selecting the similar point cloud S. ${\mathrm{NorV}}_{P}=\left[n_{P_{1}},n_{P_{2}},\ldots n_{P_{\bar{N}}}\right]$ is the normal vectors of the candidate collection. $\bar{N}$ is the number of candidates in this round. The Euclidean distance between each normal of Q and ${\mathrm{NorV}}_{P}$ is calculated, generating a distance matrix E with the size of $N^{Q}\times \sum_{i=1}^{\bar{N}}N^{P_{i}}$.

(7) $$E_{m,n}=d_{E}\langle n_{Q}^{m},n_{P_{i}}^{n}\rangle .$$

where *m* and *n* are the indices of the normal vectors of Q and ${\mathrm{NorV}}_{P}$, respectively. $d_{E}$ is the *Euclidean* distance.

(8) $$E_{m,n}^{c}=\left\{\begin{array}{cc} 1 & E_{m,n}=\min\left(E_{m,:}\right)\\ 0 & E_{m,n}\neq \min\left(E_{m,:}\right). \end{array}\right.$$

Matrix $E^{c}$ with the size of $N^{Q}\times \sum_{i=1}^{\bar{N}}N^{P_{i}}$ locates the points with the highest similarity of normal vectors. $E_{m,:}$ represents the $m_{th}$ row of E.

(9) $$N_{P}^{1}=\sum_{n=1}^{N^{P_{1}}}E_{:,n}^{c}.$$

(10) $$N_{P}^{j}=\sum_{n=\sum_{i=1}^{j-1}N^{P_{i}}+1}^{\sum_{i=1}^{j}N^{P_{i}}}E_{:,n}^{c},\;j\in \left\{2,3,\ldots ,\bar{N}\right\}.$$

where $E_{:,n}^{c}$ represents the $n_{th}$ column of $E^{c}$. $N_{P}^{j}$ counts the number of points with the highest similarity in $P_{j}$. The *j*-th point cloud with the maximal value of $N_{P}^{j}$ is the final selected similar point cloud S.

### 3.3. Graph-Based Reweighted Discriminative Optimization (GRDO)

#### 3.3.1. Sequence of Regressors

Let $f_{Q}$ be the feature of Q and $D_{t+1}\in R^{p\times f}$ be a matrix mapping the feature to an update vector. Given an initial parameter vector $x_{0}\in R^{p}$, the updating process is as follows:

(11) $$x_{t+1}=x_{t}-D_{t+1}\times f_{Q}.$$

The update process ends until $x_{t+1}$ converges to a stationary point, and the sequence of regressors $D_{t+1},t=0,1\ldots$ are learned through approximating the estimated parameter vector $x_{t+1}^{i}$ to the ground truth $x_{*}^{i}$.

(12) $$\begin{array}{cc} D_{t+1} & =\min_{\hat{D}}\frac{1}{N}\sum_{i=1}^{N}{∥W_{t}\left(x_{t+1}^{i}-x_{*}^{i}\right)∥}_{2}^{2}\\ & =\min_{\hat{D}}\frac{1}{N}\sum_{i=1}^{N}{∥W_{t}\left(x_{t}^{i}-\hat{D}\times f_{Q}-x_{*}^{i}\right)∥}_{2}^{2}. \end{array}$$

where *N* is the number of point clouds that participate in the training process, $x_{t}^{i}$ is the parameter vector of the *i*-th point cloud at the *t*-th iteration. $W_{t}\in R^{p\times p}$ is a weighting diagonal matrix. The detailed explanation of (12) has been provided in [11]. For simplicity, we denote $x_{t}^{i}$ as $x_{t}$ for any point cloud.

#### 3.3.2. Design the Feature $f_{Q}$

**Figure 5:**
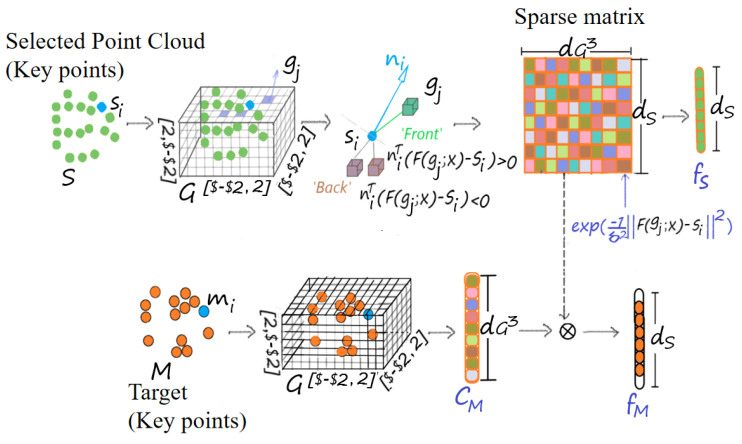
The process of feature extraction.

Good registration occurs when the surfaces of two shapes are aligned [10]. To achieve such registration, we design a feature function $h_{Q}$ to encode the relative position information of key points, making GRDO learn $D_{t+1}$ in the direction that aligns surfaces, as shown in Figure 5. We quantize the space around M into a uniform grid G spanning $\left[-2,2\right]$ in each dimension and denote a grid as $g_{j}$. Let $n_{i}$ be the normal vector of the key point $m_{i}$ in M, computed from the local plane fitted by its six neighboring points; $g^{+}=\left\{g_{j}:n_{i}^{T}\left(g_{j}-m_{i}\right)>0\right\}$ be the set of grids on the ‘front’ of $q_{i}$; and $g^{-}=\left\{g_{j}:n_{i}^{T}\left(g_{j}-m_{i}\right)<0\right\}$ contains the remaining grids. We design a sparse matrix $S_{p}$ to store the relative position information between the uniform grid G and M.

(13) $${\left[S_{p}\right]}_{i,j}^{+}=\left\{\begin{array}{cc} \exp\left(-\frac{1}{\sigma^{2}}{∥g_{j}-m_{i}∥}^{2}\right) & g_{j}\notin g^{+}\\ 0 & g_{j}\in g^{+} \end{array}\right.$$

(14) $${\left[S_{p}\right]}_{i,j}^{-}=\left\{\begin{array}{cc} \exp\left(-\frac{1}{\sigma^{2}}{∥g_{j}-m_{i}∥}^{2}\right) & g_{j}\notin g^{-}\\ 0 & g_{j}\in g^{-} \end{array}\right.$$

(15) $$\begin{array}{cc} S_{p} & =\left[{\left[S_{p}\right]}_{i,j}^{+};{\left[S_{p}\right]}_{i,j}^{-}\right]\\ i & =1,\ldots ,d_{M},\;j=1,\ldots ,d_{G}^{3}. \end{array}$$

where σ controls the width of the *exp* function, and $d_{M}$ is the number of key points in M.

We introduce a function F that applies rigid transformation with parameter x to the source point cloud Q. $F\left(Q;x\right)$ records the transformation of Q per iteration. Then, we count the number of key points in the transformation $F\left(Q;x\right)$ that fall into each grid to form a counted vector $c_{Q}$. Then, the feature $f_{Q}$ can be calculated as follows:

(16) $$f_{Q}=c_{Q}\times S_{p}.$$

Feature $f_{Q}$ is employed to learn the sequence of regressors $D_{t+1}$ via (12). The learned regressors $D_{t+1}$ will be employed to estimate the transformation for the pair 〈S,M〉 in the transfer module.

### 3.4. Transfer Module

The transfer module intends to share the learned regressors $D_{t+1}$ with S to estimate the transformation parameter $x_{t+1}$ aligning the pair 〈S,M〉 via the following formula:

(17) $$x_{t+1}=x_{t}-D_{t+1}\times f_{S}.$$

The number of key points in the transformation $F\left(S;x\right)$ that fall into each grid forms the vector $c_{S}$. The feature of the selected point cloud $f_{S}$ can be calculated as follows:

(18) $$f_{S}=c_{S}\times S_{p}.$$

For clarity, we provide the pseudocodes for training GRDO and parameter estimation, as shown in Algorithms 1 and 2. We start by training $D_{1}$ using initial data ${\left\{\left(x_{0}^{i},x_{*}^{i}\right)\right\}}_{i=1}^{N}$, $W_{t}$, and $f_{Q}$ with (12), followed by updating $x_{1}$ with $D_{1}$ using (11). At each step, a new parameter vector can be created by recursively applying the update rule in (11). The learning process is repeated until certain termination criteria are met, for example, until the error is not reduced too much or the maximum number of iterations T is reached. Then, we count the number of key points in the transformation of S falling into each grid to form the vector $c_{S}$ and utilize the sparse matrix $S_{p}$ via (15) to obtain the feature $f_{S}$ according to (18). Finally, the learned sequence of regressors ${\left\{D_{t}\right\}}_{t=1}^{T}$ and feature $f_{S}$ are applied in (17) to estimate the transformation parameter from the selected model S to the target model M.
**Algorithm 1** Training a sequence of update maps**Require:** ${\left\{\left(x_{0}^{i},x_{*}^{i}\right)\right\}}_{i=1}^{N}$, *T*, δ, Q **Ensure:** ${\left\{D_{t}\right\}}_{t=1}^{T}$1:**for** t=0 to T−1 **do**2:    Compute $W_{t}$ according to [11]3:    Compute $f_{Q}$ with (16)4:    Compute $D_{t+1}$ with (12)5:    **for** i=1 to *N* **do**6:          Update $x_{t+1}^{i}:=x_{t}^{i}-D_{t+1}f_{Q}$7:    **end for**8:**end for**

**Algorithm 2** Parameter estimation**Require:** $x_{0}$, ${\left\{D_{t}\right\}}_{t=1}^{T}$, δ, S **Ensure:** $x_{T}$1:Count the number of key points in S falling into each grid to form $c_{S}$2:**for** t=0 to T−1 **do**3:    Compute $S_{p}$ with (15)4:    Compute $f_{S}$ with (18)5:    Update $x_{t+1}:=x_{t}-D_{t+1}f_{S}$6:**end for**

## 4. Experimentation

**Figure 6:**
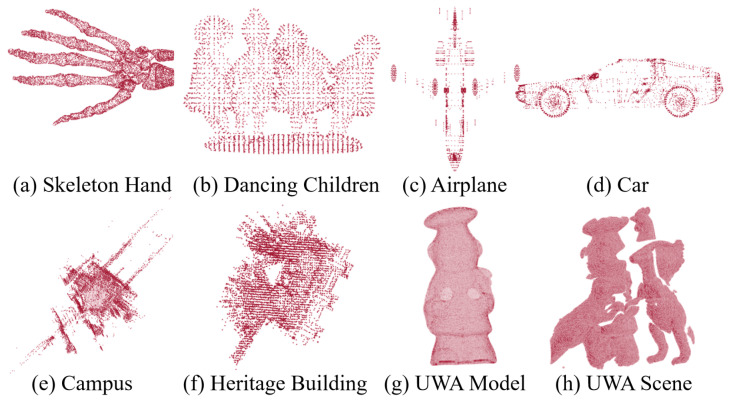
The 3D registration datasets.

This section describes applying the proposed framework SGRTmreg for the registration of multiple point cloud pairs. Three registration experiments are conducted: (1) The comparison with traditional registration methods—DO [42], RDO [11], BCPD [27], LSGCPD [28], and TEASER++ [29] on synthetic datasets (http://visionair.ge.imati.cnr/ (accessed on 25 October 2020)) [48] (in Figure 6a,b) to show the accuracy and robustness of GRDO. (2) The comparison with deep learning registration methods—FMR [18], DeepRGM [19], RPMNet [20], and RGM [21] on the ModelNet40 datasets [49] (in Figure 6c,d), which involves the selection of a similar point cloud and parameter transfer, and aims to showcase the efficacy of SGRTmreg on the registration of multiple point cloud pairs. (3) The comparison with traditional and deep learning registration methods on the WHU-TLS (Terrestrial Laser Scanner) dataset [22] (in Figure 6e,f). (4) The comparison with traditional and deep learning registration methods on the range-scan UWA dataset [23] (in Figure 6g,h) to demonstrate the registration capability of GRDO on real-world datasets.

### 4.1. Experimental Design

We normalize each point cloud $P_{i}$, the target point cloud M, and the source point cloud Q to ${\left[-1,1\right]}^{3}$. The normalized Q and the normalized $P_{i}$ are compared to select the similar point cloud S via the searching scheme (Section 3.2). We register Q and M to learn the regressors $D_{t+1}$ in the training process of GRDO. Then, the learned regressors $D_{t+1}$ are utilized to register S and M.

#### 4.1.1. GRDO Training

The parameters in the training process are similar to those in DO [42]. Given the source model Q and the target model M, we first normalized them to lie in [−1,1]. Then, we applied the following perturbations to the source model Q to generate the training samples: *(i) Rotation and Translation:* The rotation is within 45° and the translations is in ${\left[-0.3,0.3\right]}^{3}$, which represents the ground truth ($x_{*}$ in (12)). *(ii) Noise and Outliers:* Gaussian noise with the standard deviation 0.05 is added to Q; 0 to 300 points within ${\left[-1.5,1.5\right]}^{3}$ are added as the sparse outliers. A Gaussian ball of 0 to 200 points with a standard deviation of 0.1 to 0.25 simulates the structured outliers. *(iii) Occlusion:* We remove 40% to 90% points from Q to simulate occlusions [42]. We generate 30,000 training samples, and set $x_{0}$ as $0^{6}$, (N = 30,000, $x_{0}$ = $0^{6}$ in Equation (12)). Please note that the rotation range in the above settings covers the relative position of the target model M and the source model Q.

#### 4.1.2. Evaluation Metrics

Mean Square Error (MSE) evaluates the performance of registration methods, which measures the average squared difference between the coordinates of the registered point cloud and the target point cloud. Since DO, RDO, BCPD, LSGCPD, GRDO, and TEASER++ are all implemented in MATLAB 2022b, the computation time in seconds serves as an additional metric for assessing these registration methods.

#### 4.1.3. Parameter Settings

For *DO* and *RDO*, we set $\sigma^{2}$ as 0.03. The value of the tolerance of the absolute difference between the current estimation and ground truth in iterations is $1\times 10^{-4}$. For *BCPD*, the expected percentage of outliers is 0.1, the parameter in the Gaussian kernel is 2.0, and the expected length of the displacement vector is 400. For *LSGCPD*, the expected percentage of outliers is 0.1, and the maximum iteration is 30. For *TEASER++*, Graduated Non-Convexity (GNC) [50] is used to estimate rotation, and the factor for increasing/decreasing the GNC function control parameter is set to 1.4. All deep learning networks are trained on a Nvidia Geforce 2080Ti GPU with 12 G memory. The parameter settings for FMR, RGM, DeepGMR, and RPMNet are shown in Table 1.

#### 4.1.4. Registration Experiments

**Registration on synthetic datasets.** The source model Q is downsampled by selecting ∼1500 points to generate model S. The performance is evaluated under various perturbations: *(1) Rotation:* The initial angle is 0°, 30°, 60°, 90°, 120°, and 150° [default = 0° to 45°]. *(2) Noise:* The standard deviation of Gaussian noise is set to 0, 0.02, 0.04, 0.06, 0.08, and 0.1 [default = 0]. *(3) Outliers:* We set the number of outliers to 0, 100, 200, 300, 400, and 500, respectively [default = 0]. *(4) Occlusion:* The occlusion ratio is set to 0, 0.15, 0.30, 0.45, 0.60, and 0.75 [default = 0]. The random translation of all generated scenes is within ${\left[-0.3,0.3\right]}^{3}$. When one parameter is changed, the values of other parameters are fixed to the default value. We will test 750 test samples in each variable setting.

**Figure 7:**
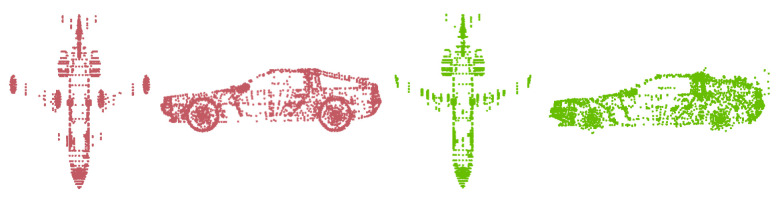
The search results for the given source models. Red shows the given source point clouds. Green shows the selected similar point clouds.

**Registration on the ModelNet40 dataset.** The ModelNet40 dataset contains pre-aligned shapes from 40 categories, split into 9843 for training and 2468 for testing. We randomly select one instance from the testing sets of two categories (Airplane and Car) as the given source models Q. Similar models S are selected from the training sets of these two categories via the proposed searching scheme. Figure 7 shows the selected similar point cloud (green) for the given point clouds (red). The perturbation settings on the ModelNet40 dataset are similar to those on synthetic datasets.

**Registration on the WHU-TLS and UWA datasets.** The WHU-TLS dataset comprises 115 scans and over 1740 million 3D points collected from 11 different environments with point density, clutter, and occlusion variations. The perturbation settings on the WHU-TLS dataset are similar to those on synthetic datasets. We uniformly sample from the original model with the replacement of almost 8000 points to generate the model Q. The UWA dataset contains 50 cluttered scenes with five objects taken with the Minolta Vivid 910 scanner in various configurations. All objects are heavily occluded (60% to 90%). From the original model of the object (chef), ∼400 points are sampled using *pcdownsample* to generate the model Q. We also downsample the scene to ∼1000 points to generate the model M. We initialize M from 0 to 45 degrees from the ground truth orientation with random translation within ${\left[-0.3,0.3\right]}^{3}$.

### 4.2. Experimental Results and Discussion

#### 4.2.1. Registration on Synthetic Datasets

**Figure 8:**
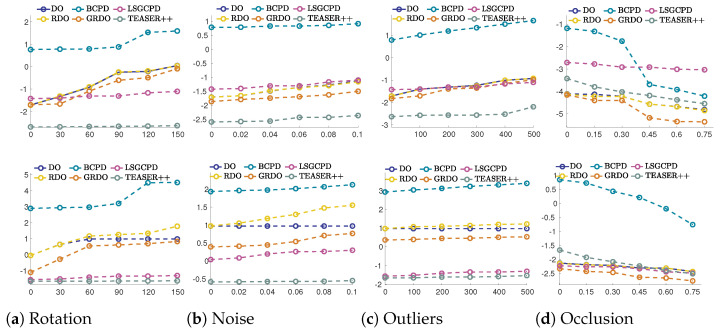
The $log_{10}$ computation time of traditional registration methods on synthetic datasets (Top—Skeleton Hand, Bottom—Dancing Children). Each *X*-axis represents varying degrees of perturbation: rotation angles, standard deviation of noise, number of outliers, and occlusion rate.

Figure 8 presents the computation time of traditional methods on synthetic datasets. (Top) and (Bottom) display the $\log_{10}$ computation time on the Skeleton Hand model and the Dancing Children model, respectively. (Left) shows that the computation time of learning-based methods (DO, RDO, and GRDO) takes longer as the rotation angle increases. Nevertheless, GRDO exhibits shorter computation time compared to DO and RDO. This is because GRDO extracts features from a limited number of key points, leading to less time to recount the number of key points falling into each grid. In contrast, BCPD needs more computing time. Meanwhile, the TEASER++ algorithm stands out as the most time-efficient method, even when dealing with large rotations. The time advantage of TEASER++ stems from its adoption of GNC for rotation estimation without solving the large-scale semidefinite programming problem. (Second and Third) show that GRDO still takes less computation time to achieve registration under various noises and outliers than DO and RDO. (Right) illustrates that all methods require less computation time as the occlusion ratio increases. However, the decline in computation time is particularly noticeable for GRDO, BCPD, and TEASER++.

Table 2 and Table 3 present the MSE of the registration results on Skeleton Hand and Dancing Children models under various perturbations, respectively. We analyze the MSE distribution via two box-plot factors (Maximum and IQR—Interquartile Range). A smaller maximum value indicates higher registration accuracy, while a smaller IQR signifies greater performance stability. The tables show the minimal maximum registration error in bold and the minimal IQR value in italics. The results highlight that BCPD and GRDO exhibit superior stability compared to other methods. Also, the registration accuracy of GRDO is the highest, especially when handling the registration with various noise and outliers.

#### 4.2.2. Registration on the ModelNet40 Dataset

**Figure 9:**
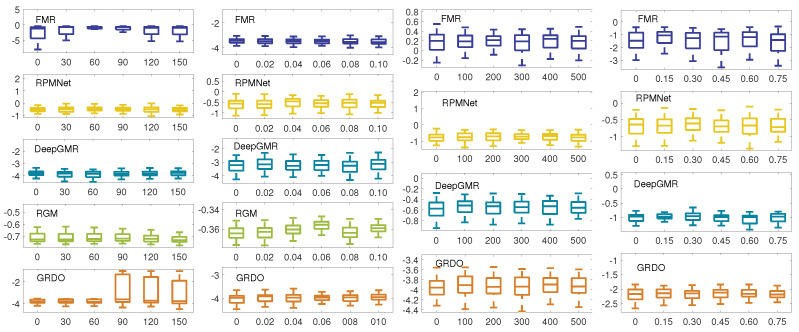

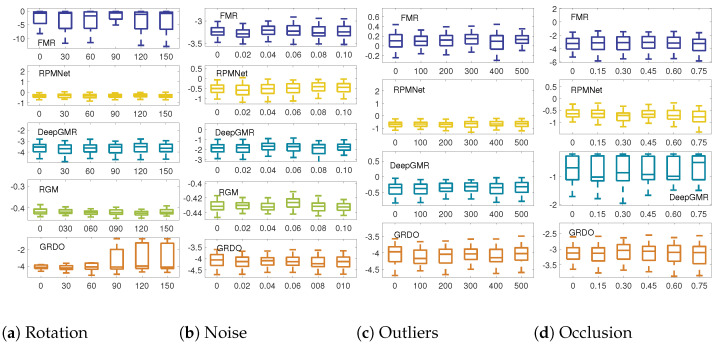
The $log_{10}$ MSE of registration results on the ModelNet40 dataset (Top—Airplane, Bottom—Car) under various perturbations. Each *X*-axis represents varying degrees of perturbation, namely rotation angles, standard deviation of noise, number of outliers, and occlusion rate.

Figure 9 shows the comparison with deep learning methods on the ModelNet40 dataset. The top and bottom show the registration results on the airplane and car models, respectively. Because RGM requires the same size of point clouds to be matched, RGM is unsuitable for registrations involving outliers or occlusions. Hereby, the performances of GRDO, FMR, RPMNet, and DeepGMR are compared. RPMNet and RGM show lower registration accuracy under various rotations. GRDO struggles with accuracy and stability for larger rotations (90° and above), while DeepGMR excels in these scenarios. Additionally, GRDO demonstrates robustness to noise and outliers, outperforming FMR. When dealing with different degrees of occlusions, RPMNet is the least accurate, while GRDO maintains high accuracy and stability.

#### 4.2.3. Registration on the WHU-TLS Dataset

**Figure 10:**
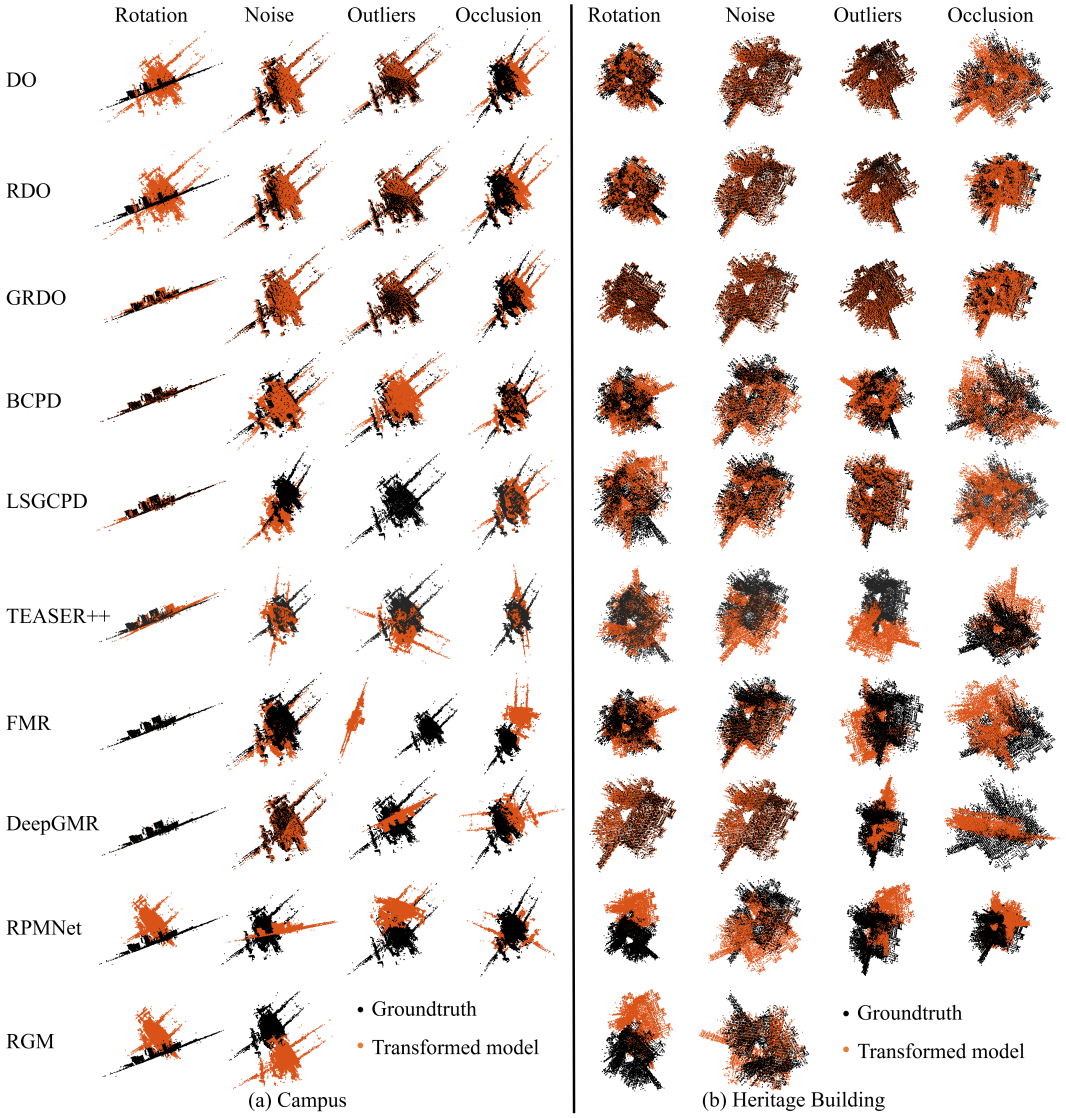
The registration results on the WHU-TLS dataset (Top—Campus, Bottom—Heritage Building). Each column shows the registration results under a specific perturbation, while each row displays the registration results of different methods.

Figure 10 displays the registration results on Campus and Heritage Building under the following perturbations: rotation—90°, noise—std=0.08, outliers—400, missing ratio—0.60. It can be seen that DeepGMR and GRDO demonstrate higher accuracy in registration when the rotation angle is 90°. When the standard deviation of Gaussian noise is 0.08, DeepGMR, GRDO, RDO, and DO perform better. Regarding the registration with outliers, LSGCPD, GRDO, RDO, and DO show superior performance. GRDO consistently maintains high accuracy even when the occlusion ratio reaches 60%.

**Figure 11:**
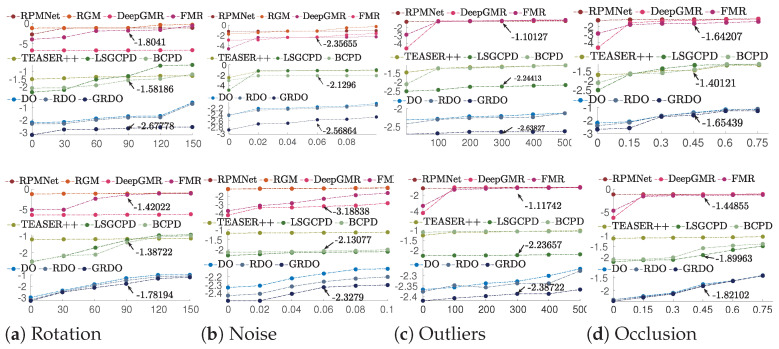
The $log_{10}$ MSE of registration results on the WHU-TLS dataset (Top—Campus, Bottom—Heritage Building). Red signifies deep learning methods, blue represents learning-based optimization methods, and green indicates traditional registration methods. Each *X*-axis represents varying degrees of perturbation: rotation angles, standard deviation of noise, number of outliers, and occlusion rate. For the sake of comparison, the comparison is marked by arrows and numeric values.

Figure 11 displays registration results on the WHU-TLS dataset under different perturbations, with the top for Campus and the bottom for Heritage Building. The red indicates the log MSE of deep learning methods, and the blue represents that of DO, RDO, and GRDO. The green shows that of BCPD, LSGCPD, and TEASER++. DeepGMR performs well with the registration under larger rotations (over 90°). DeepGMR, GRDO, and FMR demonstrate higher accuracy in achieving registration under varying degrees of noise. Traditional methods, notably DO, RDO, and GRDO, outperform deep learning methods in handling registration under outliers and occlusions.

#### 4.2.4. Registration on the UWA Dataset

**Figure 12:**
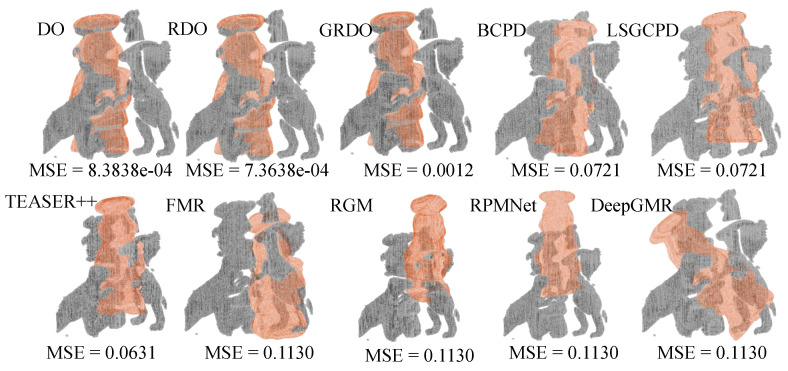
The registration results on the UWA dataset.

Figure 12 shows the registration results on the UWA dataset. Except for DO, RDO, and GRDO, other methods showcase unsatisfactory performance in registering the model and scene. RDO stands out for its accuracy. In contrast, GRDO performs poorly. GRDO is solely trained on the chef model, lacking exposure to other objects within the scene. It achieves registration using key points from both chef and scene models. Due to the body of the chef model being missing in the scene, the extracted key points from the scene graph differ significantly from those of the chef model, resulting in the poor performance of GRDO.

## 5. Discussion

### 5.1. Key Points Extraction

**Figure 13:**
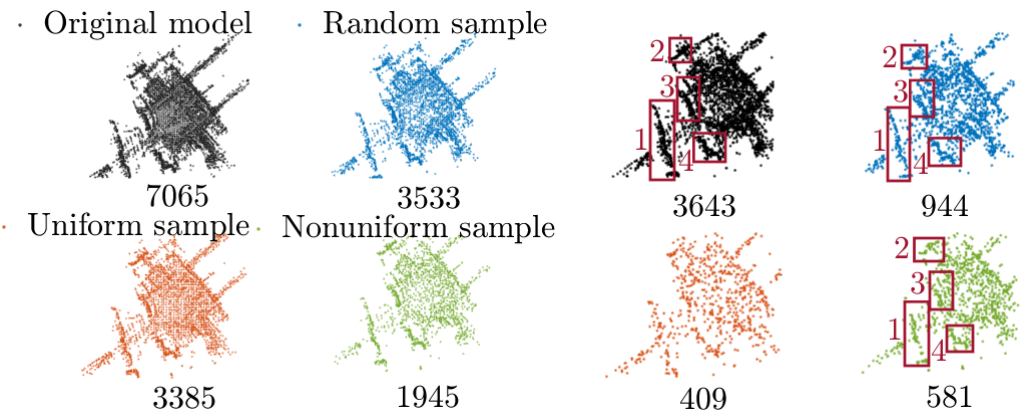
Campus model with different densities and the extracted key points. The value in the bottom-right corner represents the number of points.

We conduct experiments on the Campus model to explore the influence of point cloud density on the key point extraction. Figure 13 illustrates the point clouds with varying densities attained through random, uniform, and nonuniform downsampling, along with the extracted key points. The key points effectively capture the model shape and details (highlighted by red rectangles), as seen in Figure 13, except for those extracted using the uniform downsample method. This method merges points within the same box, averaging their locations, colors, and normals, leading to a loss of detailed information.

**Figure 14:**
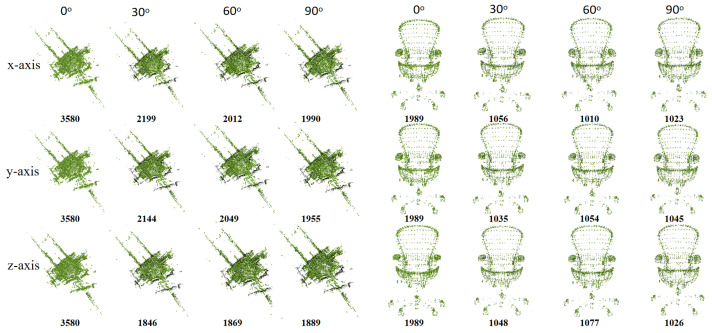
The key points extracted via Delaunay triangulation from the Campus model (WHU-TLS dataset) and the Chair model (ModelNet40 dataset) rotated at 0°, 30°, 60°, and 90° along the *X*, *Y*, and *Z* axes.

**Figure 15:**
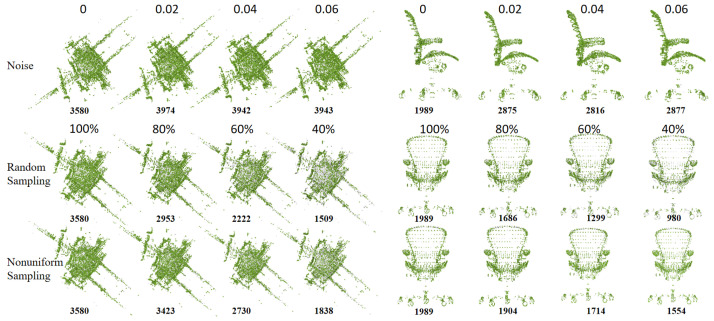
The key points extracted via Delaunay triangulation from the Campus model (WHU-TLS dataset) and the Chair model (ModelNet40 dataset) under various noise and sampling rates.

Additionally, we extract key points from point clouds with varying rotations, noise levels, and sampling rates to explore the robustness and effectiveness of Delaunay triangulation in terms of different perturbations. Figure 14 displays key points extracted via Delaunay triangulation from the Campus model (WHU-TLS dataset) and the Chair model (ModelNet40 dataset) rotated at 0°, 30°, 60°, and 90° along the *X*, *Y*, and *Z* axes. The bold black number indicates the number of key points extracted from a single point cloud, while the black points illustrate differences among key points extracted from rotated and non-rotated point clouds. It can be seen that the number of key points extracted from the point cloud rotated 90° is nearly half that of the non-rotated point cloud. For symmetric shapes like the Chair model, rotation has less impact on the performance of Delaunay triangulation, and the extracted key points adequately cover both the shape and its details in terms of various rotation angles. However, for intricate shapes like the Campus model, extracted key points generally outline the shape but overlook detailed information. As the rotation angle increases, the disparity between key points extracted from rotated and non-rotated point clouds widens, evident in the black area in the third and fourth columns. Figure 15 depicts key points extracted via Delaunay triangulation from the Campus model (WHU-TLS dataset) and the Chair model (ModelNet40 dataset) under various noise and sampling rates. The first row displays the extracted key points under Gaussian noise standard deviations of 0, 0.02, 0.04, and 0.06. The second and third rows show the extracted key points via the random sampling technique and the nonuniform sampling technique, respectively. The sampling rates are 100%, 80%, 60%, and 40%. The bold black number signifies the number of key points extracted from a single point cloud. The preservation of shape and detail highlights the robustness of the key point extraction to variations in noise and sampling.

To further explore the influence of key points extracted by Delaunay triangulation on the final registration, we rotate the Dancing Children model 30°, 60°, 90°, and 120° along the *X*, *Y*, and *Z* axes to extract their key points, while comparing the number of key points and the registration error. The number of key points and their registration error is shown in Table 4. Please note that the number of key points in this table represents the size of the intersection of the key points of the rotated model and the key points of the original model. The number of key points of the original model is 3269. It can be seen that no matter how many degrees the model is rotated, the number of key points is about 2000, which is greater than half of the number of key points of the original model. To discuss the influence of the number of key points on registration error, we also compare the MSE of registration under varying rotations between the key points and the original model. It can be seen that although the registration accuracy of the original model is higher than that of the rotated model, the gap is small. Additionally, as the degree of rotation is increased, the registration accuracy is lowered.

In summary, combining Figure 14 with Table 4, we can find that although the key points extracted by Delaunay triangulation are rotation-dependent, the shape and most details are maintained, making the gap between the registration error of the key points and that of the original model slight.

### 5.2. Searching Scheme

The proposed searching scheme comprises four screening criteria: (1) $R_{1}$—similarity in graph structure; (2) $R_{2}$—similarity in coordinates; (3) $R_{3}$—similarity in the importance of graph nodes; (4) $R_{4}$—similarity in normal vectors. To demonstrate the indispensability of screening criteria, we conduct the ablation study on MPI Dynamic FAUST [51] and ModelNet40 datasets.

**Figure 16:**
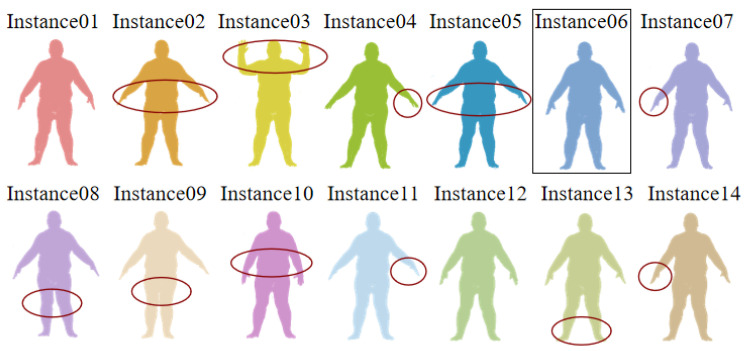
The searching instances in the MPI Dynamic FAUST dataset.

The MPI Dynamic FAUST dataset includes 10 subjects with 14 poses, and each pose contains hundreds of sequences, from which we randomly select one subject and its 14 poses as searching instances, as shown in Figure 16. We select ${\mathrm{instance}}_{6}$ (rectangle) as the reference instance Q and try to find its similar instance S from the remaining 13 instances. The ellipse shows the difference between these 13 instances and ${\mathrm{instance}}_{6}$. ${\mathrm{instance}}_{1}$ and ${\mathrm{instance}}_{12}$ are regarded as the target instances because the difference between these instances with Q is slight. Table 5 shows the result of the ablation study on the MPI Dynamic FAUST dataset. The number of candidates represents the number of instances entering the next screening round. The value after “/” represents the candidate number when $\beta^{\prime }=1$ and β=1. The value before “/” shows the candidate number when $\beta^{\prime }=3$ and β=0.80. The collaboration of these four screening criteria takes 5.538106 s to find the target instance S-${\mathrm{instance}}_{12}$. Also, we find that if the parameters are set loosely, S will not be easily eliminated.

To further explore the robustness of the searching scheme, we conduct experiments in the selected subject with its 14 poses under varying sampling and noise levels, and the Shape Distributions [52] method is used for comparison. The shape distribution quantitatively describes and compares 3D geometry using geometric characteristics evaluated by a shape function. The D2 shape distribution is renowned for its suitability in model classification and comparison. The Bhattacharyya coefficient is utilized to measure the similarity between shape distributions [53]. Given the reference instance Q and the remaining 13 candidates, we test the robustness of the proposed searching scheme using three cases: (1) Searching for a similar instance S under varying noise levels. The standard deviation of Gaussian noise is set to 13 random numbers within the range of 0 to 0.3. (2) Searching for a similar instance S under varying sampling rates. The sampling rate is set to 13 random numbers within the range of 0.7 to 1. (3) Searching for a similar instance S under varying sampling rates and noise levels, referring to the mentioned settings of sampling rate and the standard deviation. The search results are shown in Table 6. The value after “/” represents the index of the selected similar S, and the value before “/” shows the candidates with higher similarity to the given reference instance Q (${\mathrm{instance}}_{6}$). It can be seen that the proposed searching scheme is feasible for handling search tasks under various perturbations and has higher robustness than the Shape Distributions method.

**Figure 17:**
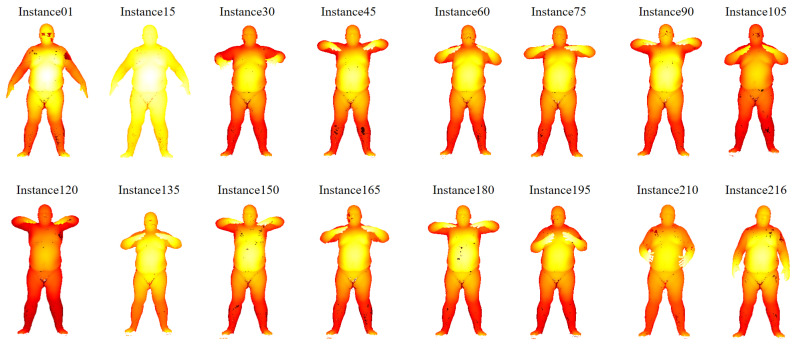
The sequence of the poses of chicken wings.

**Figure 18:**
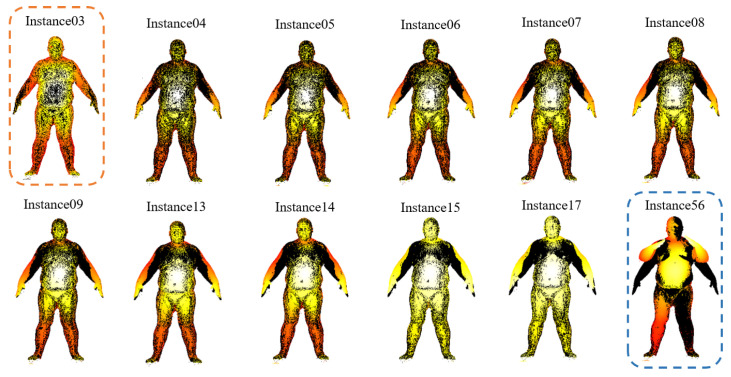
The candidates entering the second screening round and the final selected similar point clouds (dashed rectangles).

To confirm this conclusion, we randomly select one subject with a single pose (chicken wings) comprising 216 sequences with varying levels of occlusions and outliers from the MPI Dynamic FAUST datasets, as shown in Figure 17. We select ${\mathrm{instance}}_{01}$ as the reference instance Q and try to find its similar instance S from the remaining 215 instances. Table 7 shows the result of the ablation study on the MPI Dynamic FAUST dataset with the pose of chicken wings. The candidates entering the second screening round are shown in Figure 18. The black represents the reference instance ${\mathrm{instance}}_{01}$. ${\mathrm{instance}}_{56}$ is the selected similar instance S of the Shape Distributions method. It can be seen that the proposed search scheme performs better than the Shape Distribution method.

The ModelNet40 dataset contains 40 categories of CAD models, among which we select the “Car” category as the study object. The training set includes 190 instances, and the test set contains 95 instances. We randomly select ${\mathrm{instance}}_{102}$ as Q and try to find its target instance S in the remaining 284 instances. Table 8 shows the ablation study results. “∖” is used to replace the index value when the number of candidates is large. We can find that $R_{1}$ can eliminate almost one-half of instances whose graph structure is far different from that of Q, and $R_{2}$ can achieve a similar effect in reducing the number of candidates.

**Figure 19:**
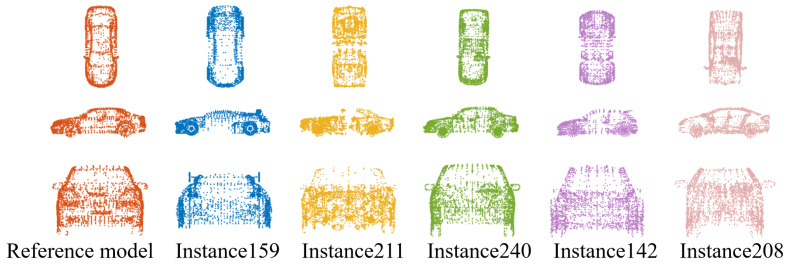
The instances from the mixed dataset entering the final screening.

In addition, we test the proposed searching scheme on objects with unseen categories using a mixed dataset comprising the MPI Dynamic FAUST dataset, ModelNet40 dataset, and SHREC’20 dataset. The SHREC’20 dataset [54] includes an elastic-stuffed toy rabbit with 11 partial scans and one full scan. For this experiment, we focus on the “Car” category from the ModelNet40 dataset with ${\mathrm{instance}}_{102}$ as the reference instance Q, resulting in a total of 310 candidate instances. We set $\beta^{\prime }=5$ and β=0.99. Table 9 shows the ablation study results. Due to $\beta^{\prime }=5$, there are five candidates entering $R_{4}$, as shown in Figure 19. It can be seen that the proposed searching scheme can locate targets in objects with unseen categories.

These four screening criteria play distinct roles in the searching scheme. $R_{1}$ filters out instances whose structure is far different from that of Q. $R_{2}$ eliminates instances with dissimilar point distributions to Q. $R_{3}$ screens instances with similar node proximity to Q. $R_{4}$ selects the most similar instance based on the normal vector. In summary, the proposed searching scheme follows a coarse-to-fine approach to efficiently search S for Q. Each of the four screening criteria is essential and complements one another. Please note that $R_{2}$ will discard similar point cloud S if the rotation angle between S and Q exceeds 75° because DPGMM clusters point clouds by coordinates in $R_{2}$.

### 5.3. GRDO

#### 5.3.1. Partial Point Cloud Registration

**Figure 20:**
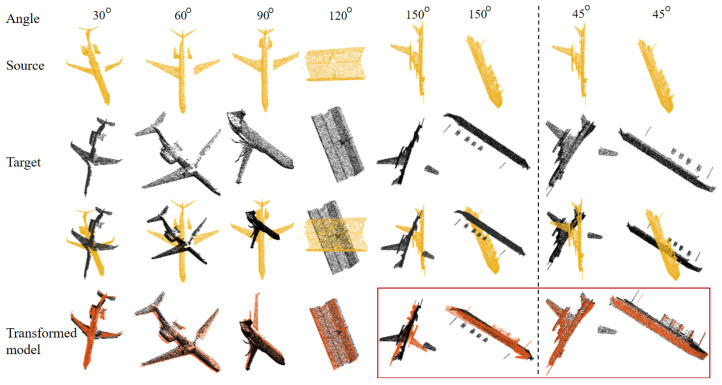
Registration on the MVP dataset with various rotation angles.

We conduct registration experiments on the MVP dataset [36] under various rotations to evaluate the performance of GRDO on partial point cloud registration. The MVP dataset is a large-scale multi-view partial point cloud dataset comprising over 100,000 high-quality scans, and it provides a training set with 62,400 partial–complete point cloud pairs and a test set with 41,800 pairs. We randomly select six pairs for registration. Notably, GRDO is solely trained on complete models, employing the following training parameters: rotation—90°, noise—0, outliers—0, and missing ratio—0.4 to 0.9. In the test stage, we use the learned regressors to register the partial point cloud pairs directly. Figure 20 shows that GRDO can register partial point cloud pairs and perform well under varying occlusions yet struggles with larger rotations.

#### 5.3.2. Different Density Distribution

**Figure 21:**
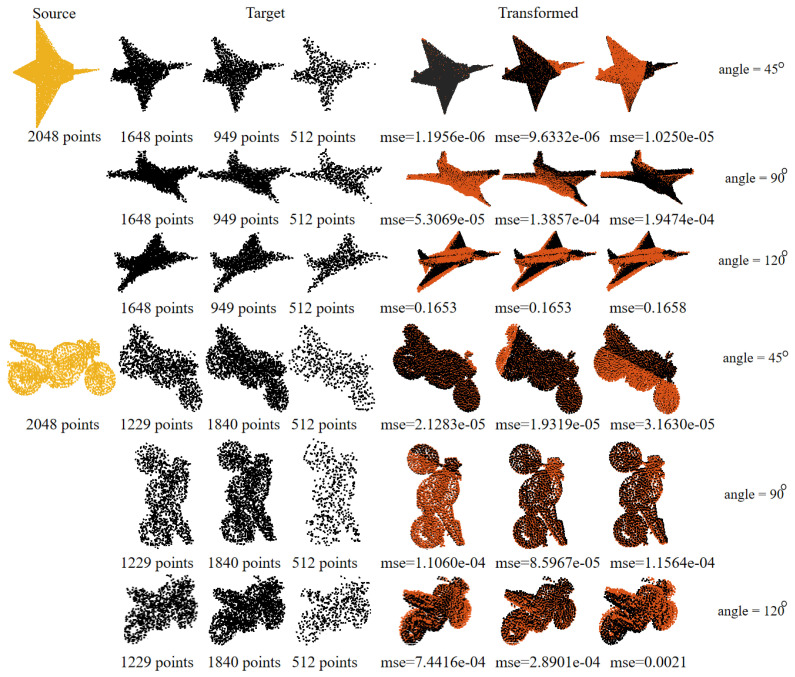
Registration on the MVP dataset with various density distributions.

Given that GRDO extracts key points based on graph structures, we suspect its sensitivity to matching point clouds with varying densities, especially when the source and target point clouds are from different or noisy density distributions. We conduct registration experiments on the MVP dataset with varying densities to investigate this. We first rotate the original model by 45°, 90°, and 120° to obtain the ground truth, then add Gaussian noise with a standard deviation of 0.02 to the ground truth, which is downsampled via the random sample, uniform sample, and nonuniform sample methods to create target models. The training parameters are rotation—150°, noise—std=0.05, outliers—0, and missing ratio—0. The registration results are shown in Figure 21. As the number of points increased, the registration accuracy improved significantly. Surprisingly, even with a threefold difference in the number of points between point clouds (2048 vs. 512), GRDO successfully registered them, proving its resilience to density distribution variations while maintaining high accuracy.

**Figure 22:**
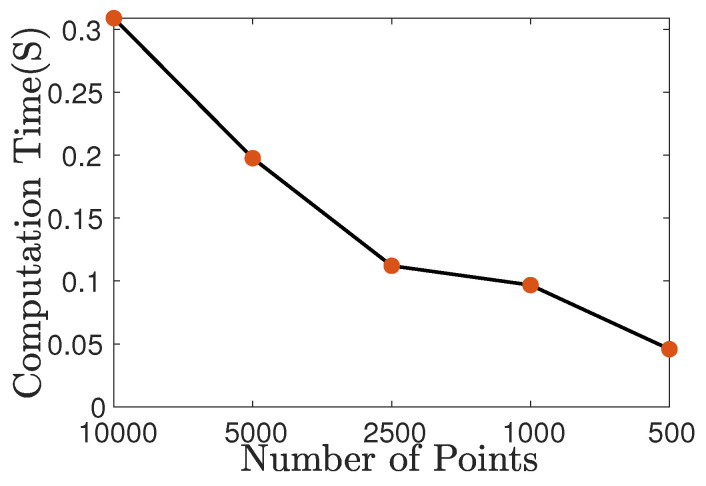
The computation time on the motorcycle model with different sizes.

To explore the influence of the number of points on computation time, we downsample the MVP dataset (motorcycle) to 10,000, 5000, 2500, 1000, and 500 points, respectively, while comparing the computation time. The computation time is shown in Figure 22. Please note that the computation time is the time for registering the model with 10,000, 5000, 2500, 1000, and 500 points to the model with 10,000 points, respectively. It can be seen that as the number of points decreases, the computation time becomes shorter.

### 5.4. Transfer Module

**Figure 23:**
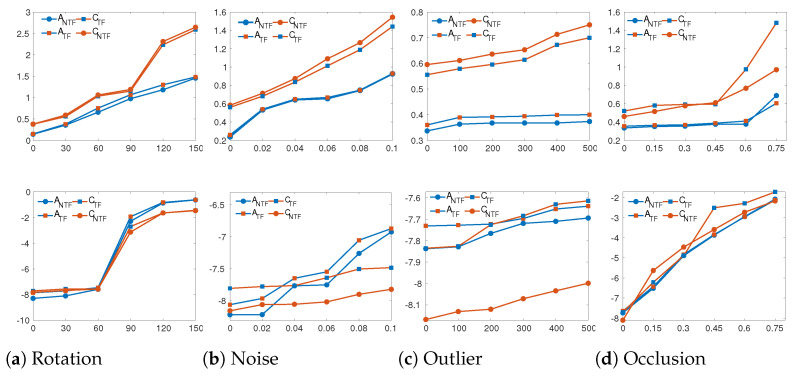
The comparison of GRDO with transfer module ($GRDO_{TF}$) and GRDO without transfer module ($GRDO_{NTF}$) under different perturbation settings on the ModelNet40 dataset (A—Airplane, C—Car). Top displays the comparison of computation time. Bottom shows the log_10_MSE. Each *X*-axis is varying degrees of perturbation, namely rotation angles, standard deviation of noise, number of outliers, and occlusion rate.

To validate the transfer module in transformation estimation, we conduct a comparative experiment on the ModelNet40 dataset between registration using $GRDO_{TF}$ (with the module) and $GRDO_{NTF}$ (without the module). Figure 23 shows the registration results on Airplane and Car models. $GRDO_{TF}$ is represented by the solid line with a square, while $GRDO_{NTF}$ is shown by the solid line with a circle. Top displays the comparison of computation time. Bottom shows the log_10_MSE. $GRDO_{NTF}$ generally has shorter computational time, better registration accuracy, and similar robustness and stability compared to $GRDO_{TF}$. Despite having lower accuracy compared to $GRDO_{NTF}$, $GRDO_{TF}$ exhibits high robustness and stability, surpassing most of the comparison methods. Thus, the transfer module is essential and highly effective for learning-based optimization in the registration of multiple point cloud pairs.

### 5.5. Comparison with Learning-Based Methods

The memory requirement is $O\left(N\left(\left(\left(c_{1}\;+\;c_{2}\right)N^{M}\;+\;c_{2}N^{S}\right)\right)\;+\;c_{3}N^{M}\times N^{M}\right)$ for learning $D_{t+1}$ in DO, which largely depends on the number of points [11]. GRDO extracts features from key points, substantially reducing the storage requirement for learning $D_{t+1}$. Compared to deep learning methods, learning-based optimization approaches (DO, RDO, and GRDO) achieve more stable and robust registration under various perturbations. Deep learning methods face challenges in converging to optimal solutions when dealing with perturbations like noises and outliers due to their data-driven nature. In contrast, model/feature-driven learning-based optimization methods excel in handling such perturbations. Although learning-based optimization methods are not as flexible as deep learning methods, SGRTmreg provides a new perspective for achieving it. A breakthrough in developing a more general feature could enable learning-based optimization methods to achieve multi-number and multi-category point cloud registration efficiently.

### 5.6. Limitations

GRDO outperforms DO and RDO in terms of computation time and storage, but it has limitations in achieving registration on model and scene. These limitations arise from the key point extraction process, which relies on the graph structure. When matching point clouds with significantly different graph structures, the performance of GRDO diminishes, making it challenging to register partial point cloud pairs with vastly different graph structures, such as large outdoor and indoor scenes. While the proposed SGRTmreg framework can achieve multiple pairwise registrations, it is limited to similar point cloud pairs due to the poor generalization of the feature extraction method, restricting the applicability of the learned regressors.

## 6. Conclusions

This paper presents SGRTmreg, a framework for the registration of multiple point cloud pairs, featuring a proficient searching scheme to find similar point clouds, the learning-based optimization algorithm GRDO for registering point cloud pairs, and a transfer module for additional registrations. The searching scheme selects a similar point cloud for a given one from a collection by using four similarity measurements: graph structure, shape, inner structure, and surface direction. Experimental results demonstrate that the searching scheme can select similar point clouds under various perturbations and from mixed datasets. GRDO learns shared regressors from key points of point clouds, enabling faster and more efficient registration. Experimental results show its high robustness and efficiency. Four experiments validate the potential of SGRTmreg, showing its high performance in point cloud registration. Compared to deep learning and learning-based methods, SGRTmreg exhibits superior accuracy, efficiency, and robustness. GRDO stands out among advanced learning-based optimization methods with reduced computational cost.

Future work includes designing a generalized parameter representation for rigid and non-rigid registration and developing a novel feature encoding method to estimate correspondences for real-world scene registration. On this basis, we anticipate applying GRDO, the searching scheme, and the transfer module to a wider range of computer graphics and computer vision tasks, such as non-rigid registration and image denoising.

## Figures and Tables

**Table 1:** Parameter settings for deep learning methods.

Method	Iteration	Optimizer	Learning Rate	Epoch	Batch Size
FMR	10	Adam	$1\times 10^{-3}$	100	32
RGM	2	SGD	$1\times 10^{-3}$	300	4
DeepGMR	1	Adam	$1\times 10^{-3}$	100	32
RPMNet	2	Adam	$1\times 10^{-4}$	1000	8

**Table 2:** The registration results on the skeleton hand ($10^{-4}$).

	DO		RDO		GRDO		BCPD		LSGCPD		TEASER++	
	Maximum	IQR	Maximum	IQR	Maximum	IQR	Maximum	IQR	Maximum	IQR	Maximum	IQR
R (90)	2.491	1.445	2.497	1.488	**1.121**	***1.261***	4.794	5.746	3351	1393	3795	3226
R (120)	2239	3339	1273	3334	**1266**	2085	3713	3371	2807	***1305***	3865	*2875*
R (150)	3789	3311	3589	3010	**3456**	2649	3773	3376	3529	***1449***	3805	*2996*
N (006)	56.00	39.00	6.205	3.151	**5.982**	***3.064***	53.00	4.931	1585	39.00	3682	2957
N (008)	59.00	62.00	6.932	5.170	**6.853**	5.112	77.00	***4.352***	1539	62.00	4060	2803
N (010)	66.00	73.00	7.418	3.988	**7.207**	***3.882***	104.0	4.536	1414	73.00	3934	2769
O (300)	9.067	7.132	5.719	3.274	**5.567**	***3.254***	742.0	355.0	1696	1114	4983	2697
O (400)	11.00	8.068	6.101	3.676	**6.084**	***3.662***	743.0	459.0	1635	1099	5234	2749
O (500)	9.792	8.566	7.713	4.180	**7.012**	***4.055***	719.0	337.0	1546	627.0	4999	2673
I (045)	491.0	497.0	**493.0**	497.0	513.0	***448.0***	1586	2772	1443	915.0	5371	2882
I (060)	1051	974.0	1046	974.0	**998.0**	***926.0***	1103	1903	1484	1059	5192	2561
I (075)	1286	1397	1283	1400	**1246**	1295	1258	2097	1492	***959.0***	5623	3075

**Table 3:** The registration results on the dancing children model ($10^{-4}$).

	DO		RDO		GRDO		BCPD		LSGCPD		TEASER++	
	Maximum	IQR	Maximum	IQR	Maximum	IQR	Maximum	IQR	Maximum	IQR	Maximum	IQR
R (90)	2684	423.0	2675	420.0	2235	263.0	**0.007**	***0.002***	3695	1381	4773	2313
R (120)	2862	350.0	2862	346.0	**2133**	***194.0***	4304	2855	3776	1483	4823	1875
R (150)	4230	2793	4125	2793	**3807**	***1090***	4283	2855	4471	1211	4310	2205
N (006)	17.00	17.00	13.00	9.000	**9.000**	9.000	35.00	**0.938**	1995	1234	4946	2058
N (008)	24.00	17.00	16.00	9.000	**12.00**	9.000	60.00	***1.710***	1779	1033	5012	2393
N (010)	25.00	17.00	19.00	12.00	**17.00**	12.00	91.00	**2.060**	1732	1047	5195	2386
O (300)	13.00	12.00	12.00	10.00	**8.000**	9.000	488.0	***9.577***	2004	1311	7283	3536
O (400)	15.00	10.00	11.00	7.000	**11.00**	7.000	616.0	***7.399***	2002	1150	6373	2470
O (500)	14.00	12.00	13.00	7.000	**11.00**	7.000	459.0	***7.464***	1939	1108	6882	2803
I (045)	**705.0**	863.0	706.0	858.0	739.0	***802.0***	1355	2281	1706	1012	6029	2534
I (060)	1455	1192	1350	1192	1389	1866	**1025**	2212	1672	***1075***	8092	3958
I(075)	2496	1696	2493	1703	2702	1210	**1296**	2368	1817	***1015***	6882	3542

**Table 4:** The number of key points (NP) and their registration error (MSE).

	Key Points								Original Model			
	30°		60°		90°		120°		30°	60°	90°	120°
	NP	MSE	NP	MSE	NP	MSE	NP	MSE	MSE	MSE	MSE	MSE
*X*-axis	1850	0.0191	1792	0.0225	1668	0.0242	1777	0.1287	0.0141	0.0271	0.0389	0.1387
*Y*-axis	1834	0.0152	1833	0.0150	1646	0.0815	1786	0.0894	0.0102	0.0178	0.0798	0.0882
*Z*-axis	1716	0.0192	1709	0.0465	1608	0.1415	1702	0.1689	0.0145	0.0468	0.1401	0.1625

**Table 5:** Result of ablation study of searching scheme on the MPI dynamic FAUST dataset.

	Number of Training Instances	Number of Candidates	Index
$R_{1}$	13	10/3	1, 2, 4, 5, 7, 9, 10, 11, 12, 14/4, 11, 14
$R_{1}$, $R_{2}$	13	7	2, 5, 7, 9, 10, 12, 14
$R_{1}$, $R_{2}$, $R_{3}$	13	3/1	10, 12, 14/14
$R_{1}$, $R_{2}$, $R_{3}$, $R_{4}$	13	1	12

**Table 6:** The search results for the MPI Dynamic FAUST dataset under varying perturbations.

	Varying Noise	Varying Sampling	Varying Noise and Sampling
Searching Scheme	10, 12, 14 /12	11, 12, 13/12	9, 10, 12/12
D2 Shape Distribution	2, 5, 9/9	1, 10, 11/11	1, 10, 11 /10

**Table 7:** Result of ablation study on the MPI dynamic FAUST dataset with the pose of chicken wings.

	Number of Training Instances	Number of Candidates	Index
$R_{1}$	215	11	3, 4, 5, 6, 7, 8, 9, 13, 14, 15, 17
$R_{1}$, $R_{2}$	215	8	3, 4, 5, 6, 7, 8, 9, 13
$R_{1}$, $R_{2}$, $R_{3}$	215	3	3, 6, 7
$R_{1}$, $R_{2}$, $R_{3}$, $R_{4}$	215	1	3

**Table 8:** Result of ablation study of searching scheme on the ModelNet40 (car) dataset.

	Number of Training Instances	Number of Candidates	Index
$R_{1}$	284	109/98	\
$R_{1}$, $R_{2}$	284	47	\
$R_{1}$, $R_{2}$, $R_{3}$	284	3/1	159, 211, 240/159
$R_{1}$, $R_{2}$, $R_{3}$, $R_{4}$	284	1	240

**Table 9:** Result of ablation study of searching scheme on the mixture of datasets.

	Number of Training Instances	Number of Candidates	Index
$R_{1}$	310	100	\
$R_{1}$, $R_{2}$	310	47	\
$R_{1}$, $R_{2}$, $R_{3}$	310	5/1	142, 159, 208, 211, 240
$R_{1}$, $R_{2}$, $R_{3}$, $R_{4}$	310	1	240

## Data Availability

Synthetic datasets: http://visionair.ge.imati.cnr/ (accessed on 25 October 2020); ModelNet40: https://paperswithcode.com/dataset/modelnet (accessed on 6 April 2022); WHU-TLS dataset: https://www.bing.com/search?q=whu-tls+dataset&qs=UT&pq=+whu-tls+dataset&sc=10-16&cvid=EC379475CC9C4D4AB89CC94AAB98E958&FORM=QBRE&sp=1&lq=0&sm=csrmain (accessed on 9 April 2022); MPI Dynamic FAUST dataset: https://dfaust.is.tue.mpg.de/ (accessed on 18 February 2024); MVP dataset: https://mvp-dataset.github.io/ (accessed on 23 February 2024).
